# Impact of Τh1 and Τh2 cytokines in the progression of idiopathic nephrotic syndrome due to focal segmental glomerulosclerosis and minimal change disease

**DOI:** 10.15171/jnp.2017.32

**Published:** 2016-12-25

**Authors:** Maria Stangou, Μichael Spartalis, Dimitra-Vasilia Daikidou, Theodora Kouloukourgiotou, Erasmia Sampani, Ioanna-Theologia Lambropoulou, Afroditi Pantzaki, Αikaterini Papagianni, George Efstratiadis

**Affiliations:** ^1^Department of Nephrology, Aristotle University of Thessaloniki, Hippokration Hospital, Thessaloniki, Greece; ^2^Department of Pathology, Hippokration Hospital, Thessaloniki, Greece Original Article

**Keywords:** Nephrotic syndrome, Cytokines, Glomerulosclerosis, Tubular atrophy

## Abstract

**Background:**

Differential diagnosis between primary focal segmental glomerulosclerosis (FSGS) and minimal change disease (MCD) is sometimes difficult as nephrotic syndrome is the main clinical symptom in both diseases.

**Objectives:**

This study has attempted to evaluate the urinary excretion of Th1 and Th2 cytokines as potential biomarkers in distinguishing the two types of nephrotic syndrome, and predicting outcome of renal function.

**Patients and Methods:**

Thirty-six patients with FSGS (M/F 22/14, Age; 41.9 ± 17 years, SCr=1.7 ± 0.8 mg/dL, UProt=4.7 ± 5.5 g/24 h), and 21 with MCD (M/F 5/16, Age; 41.4 ± 15 years, SCr = 1 ± 0.4 mg/dL, UProt = 7.9 ± 9.3 g/24 h) were included in the study. Τh1 (IL-2, IL-12, GM-CSF, INF-γ, TNF-α) and Th2 cytokines (IL-4, IL-5, IL-10, IL-13) were measured by multiple cytokine assay, Luminex technology, in first morning urinary samples collected at the day of renal biopsy.

**Results:**

No significant differences in urinary excretion of all cytokines were found between FSGS and MCD patients. In FSGS however, IL-12 urinary levels were independent factor correlated with both global sclerosis (R = 0.5, *P* = 0.009) and interstitial fibrosis (R = 0.5, *P* = 0.02). Th1 cytokines (IL-2 and GM-CSF) were significantly increased in FSGS patients who did not respond to treatment (*P* = 0.03 and *P* = 0.007, respectively). Th2 cytokines (IL-4, IL-5, IL-10, IL-13) were significantly increased in MCD patients with frequent relapses (*P* = 0.05, *P* = 0.001, *P* = 0.01, *P* = 0.03).

**Conclusions:**

Urinary excretion of Th1 and Th2 cytokines cannot discriminate FSGS from MCD. Th1 cytokines, especially IL-12, IL-2 and GM-CSF, may be involved in pathology and progression of FSGS, while Th2 cytokines are implicated in frequent relapses of nephrotic syndrome in MCD.

**Implication for health policy/practice/research/medical education:**

Many different glomerular diseases can present as nephrotic syndrome, the most common being FSGS and MCD. In clinical practice very often it is becoming difficult to differentiate between these two primary glomerulopathies, as histology of FSGS includes focal and segmental lesions, that can be missed, and histology of MCD consists of podocyte injury, which is common in both disorders. Many efforts have been made to define serum or urinary biomarkers which may help in the differentiation of FSGS and MCD. In this study we evaluated the urinary excretion of Th1 and Th2 cytokines and found that although urinary excretion of the cytokines could not discriminate between two entities, they had different roles and could predict renal function. Th1 cytokines seem to participate in histology and outcome of FSGS, while Th2 cytokines play significant role in MCD.

## 1. Background

Primary focal segmental glomerulosclerosis (FSGS) and minimal change disease (MCD) are the two main types of glomerulopathy associated with nephrotic syndrome (1-3). The two diseases share many clinical and histologic findings at presentation. Nephrotic syndrome (NS) and microscopic hematuria are the main clinical features present at time of diagnosis. Even the impairment of renal function, although most frequently seen in FSGS may also present in MCD, as acute renal failure. Furthermore, common histological finding is podocyte injury, including fusion of foot processes with or without podocyte hypertrophy and hyperplasia. Histological lesions in FSGS are by definition focal and segmental in nature (3-5). This type of lesion can be missed in renal biopsy, particularly with a small kidney core, and FSGS can be misdiagnosed (3). However, the two diseases have totally different response to treatment, especially to steroids, and different long-term outcome (6,7). The identification of biomarkers in NS would be extremely useful, in many possible ways, including distinction between FSGS and MCD, discrimination of FSGS subtypes (FSGS not-otherwise specified, hilar FSGS, tip-lesion, collapsing, cellular), estimation of response to treatment and renal outcome, prediction of recurrence of FSGS after completing treatment, and after transplantation (8,9). Metalloproteinase, growth factors, such as epidermal growth factor (EGF) and transforming growth factor (TGF-β1), a1-antitrypsin, fragments of albumin and Tamm-Horsfall protein and miRNAs are few examples of the molecules whose urinary levels can be important in discriminating FSGS and MCD and also give information about disease outcome (9-13).

Cytokines produced by Th1 and Th2 lymphocytes have extensively been studied in autoimmune diseases. Th1 cytokines including INF-γ, TNF-α, IL-2, IL-12 and IL-23, are produced mainly by Th1 lymphocytes, in response to microbes, intracellular parasites, viruses and in autoimmune reactions. Excessive Th1 cytokine responses in an effort to face microbial infection and control autoimmune reactions, can lead to uncontrolled tissue damage (14). Th2-lymphocytes are primarily made in response to helminths (worms) and extracellular microbes and also after stimulation by allergens, and toxins. The Th2-type cytokines include IL-4, IL-5, IL-13, and IL-10, and are responsible for anti-inflammatory reactions, including eosinophil and B-cell activation, IgE production and construction of antibodies (15). In a healthy environment the pre-inflammatory reactions of Th1 cytokines as the main contributors in cellular immunity are balanced by the anti-inflammatory activity of Th2 cytokines, the principal actors in humoral immunity (16,17).

## 2. Objectives

The aim of the present study was to investigate whether urinary Th1/Th2 cytokine levels may be useful in the differential diagnosis between FSGS and MCD, and also their possible value as representatives of renal histology and predictors of renal function outcome.

## 3. Patients and Methods

### 3.1. Patients

Fifty-seven patients who presented with nephrotic syndrome and diagnosed as FSGS or MCD were included in the study. The period of patient enrolment was from January 2004 till January 2014. All patients were referred, diagnosed and followed to the department of nephrology, Aristotle University of Thessaloniki, Hippokration Hospital.

Inclusion criteria were: 1) age>18 years, 2) nephrotic syndrome at presentation, 3) renal biopsy showing FSGS or MCD, 4) no previous treatment with steroids, and immunosuppressants.

Exclusion criteria were: 1) Patients with proven or suspected secondary FSGS on renal biopsy, obese or overweight patients, patients with chronic hypertension, systemic diseases (diabetes mellitus), infections (HIV, HBV, HCV), or drug abuse; 2) patients who developed FSGS on the top of a known type of primary glomerulonephritis (for example FSGS on the top of membranous nephropathy or IgAN); and 3) Patients on chronic use of Nonsteroidal anti-inflammatory drugs (NSAIDs).

All participants were consented before entering the study. After their discharge from hospital, patients were followed up regularly, on an out-patient basis, every three months for the first year and every 3-6 months for the rest of follow-up period, totally 56.25 (12-120) months. Patients were censored if they reached end-stage renal disease (ESRD) and started on hemodialysis.

### 3.2. Assessment of kidney biopsies

The diagnosis of FSGS and MCN was based on optical microscopy, immunofluorescence and/or immunohistochemistry, and electron microscopy in selected cases. Renal biopsies were re-evaluated by our pathologist estimating the percentage of global sclerosis, presence of mesangial hyperplasia, endocapillary hypercellularity, and severity of tubular atrophy, interstitial infiltration and arteriosclerosis. Mesangial and endocapillary hyperplasia were estimated as dichotomous variables (M0-M1, E0-E1), while the degree of tubular atrophy, interstitial infiltration and arteriosclerosis were graded in a scale of 0-2, were 0 meant absent, 1 moderate and 2 severe lesions.

### 3.3. Measurement of urinary cytokines

Urine samples were collected under sterile conditions at the day of renal biopsy. Ten healthy individuals were used as controls. First morning middle stream urine samples were immediately centrifuged at 1500 rpm, for 10 minutes and the supernatant was aliquoted and kept in -70°C, until assayed. Bio-Plex human cytokine assay, using Luminex technology, was applied for the measurement of Th1 (IL-2, IL-12, GM-CSF, INF-γ, TNF-α) and Th2 (IL-4, IL-5, IL-10, IL-13). All cytokines were measured simultaneously in the same urinary sample. Premixed beads coated with target capture antibodies were transferred to each well of a flat filter plate and washed twice with Bio-Plex wash buffer. After incubation with the samples and premixed detection antibodies, the beads were resuspended in Bio-Plex assay buffer and read on the Bio-Plex suspension array system. Data were analysed using Bio-Plex Manager™ software with 5PL curve fitting. Midstream morning urine samples obtained from 20 healthy individuals were used as controls. Urinary cytokine levels were normalised for urinary creatinine (UCr) concentration and were expressed as pg/mg UCr.

### 3.4. Definitions

Estimated glomerular filtration rate (eGFR ) was calculated based on CKD-EPI formula.

Total response to treatment was defined as a reduction in proteinuria <0.3 g/24 h, with normal levels of serum albumin (>3.5 g/L) and maintaining of renal function to previous levels. Partial response was regarded the reduction of proteinuria by ≥50% of initial levels, but still remaining within 0.3-3.5 g/24 h and stable renal function (change in serum creatinine <25%). Patients with deteriorating renal function and/or no reduction in proteinuria were considered as non-responders.

Increase of proteinuria to levels ≥3.5 g/24 h, in patients with previous partial or total response, was considered as relapse.

### 3.5. Ethical issues

The project was done with consideration of ethical issues and obtaining license from the ethics committee of university and obtaining the written consent of participants. The study was done according to ethical standards of human experimentation in accordance to the Helsinki Declaration.

### 3.6. Statistical analysis

All values were expressed as mean ± standard deviation (SD). Statistical analysis was performed using SPSS 17.0 for Windows. Pearson’s and Spearman’s coefficients were used for the correlation between parametric and non-parametric variables respectively. Multivariate stepwise analysis was performed to estimate the independent parameters correlated with the outcome of renal function. Differences between groups were estimated by Mann-Whitney U test. Kaplan-Meyer with log-rank (Mantel-Cox) test was performed to estimate renal survival according to histology and urinary cytokines levels. *P* values of <0.05 were considered as statistically significant.

## Results

### 4.1. Clinical, laboratory and histological findings at time of diagnosis

Thirty-six patients with FSGS, (M/F 20/16, mean age 41 [range 16-69] years) and 21 patients with MCD (M/F 10/11, mean age 41 [18-62] years) participated in the study and their laboratory and pathology findings are depicted on Table 1. All patients had nephrotic syndrome, with no significant differences between the two groups in the levels of proteinuria. Microscopic hematuria was more common and renal function was significantly impaired in patients with FSGS, as was the severity of histological lesions.

**Table 1:** Clinical, laboratory and histological findings in patients with FSGS and MCN

	**FSGS** **n=36**	**MCD** **n=21**	**P**
M/F	20/16	10/11	NS
Age (years)	41 (16-69)	41 (18-62)	NS
MBP	103.3±6.2	90±7.2	NS
Microscopic hematuria (%)	30 (83.3)	8 (38)	0.001
SCr (mg/dL)	1.8±0.8	0.9±0.4	0.001
eGFR (mL/min)	48.7±27	84.4±30	0.001
Salb (g/dl)	3.5±0.7	2.9±1.2	NS
UProt (g/24 h)	4±2.5	5.1±2.8	NS
Histology			
Global sclerosis (%)	29±23	1.5±3.2	0.0001
Mesangial hyperplasia	0.6±0.8	0.2±0.4	NS
Endothelial hypertrophy	0.3±0.4	0	0.005
TIN fibrosis	1±0.7	0.5±0.5	0.02
Arteriosclerosis	1±0.6	0.4±0.5	0.006

### 4.2. Urinary cytokine excretion. Differences between FSGS and MCN and correlation with histology

**Figure 1:**
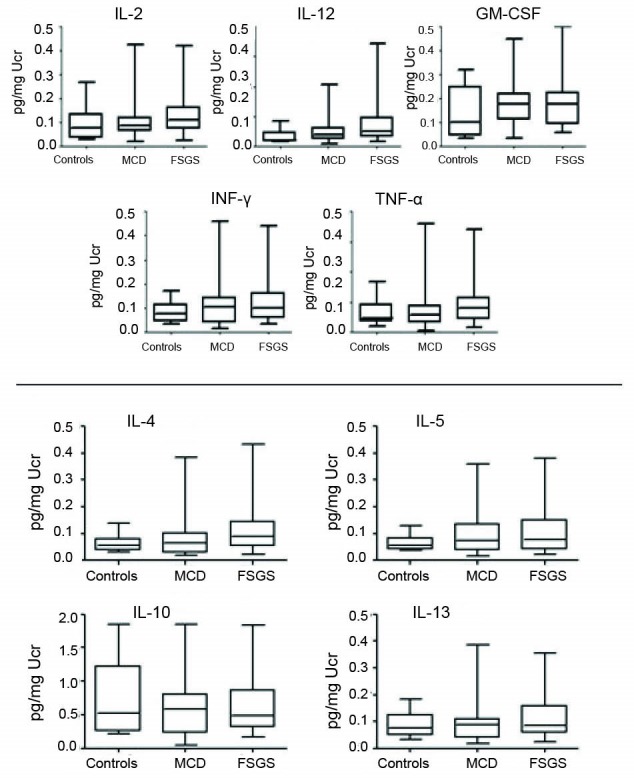
Th1 and Th2 cytokine urinary excretion in MCD and FSGS patients. (A) Urinary Th1 cytokines in patients with MCD and FSGS; (B) Urinary Th2 cytokines in patients with MCD and FSGS.

The urinary levels of Th1 (IL-2, IL-12, GM-CSF, INF-γ, TNF-α) and Th2 (IL-4, IL-5, IL-10, IL-13) in FSGS and MCD patients are shown in Figure 1.

Urinary levels (pg/mg UCr) of Th1 cytokines in FSGS were; IL-2: 0.24 (0.05-0.83), IL-12: 0.15 (0.03-0.68), GM-CSF: 0.37 (0.1-1), INF-γ: 0.12 (0.03-0.44), TNF-α: 0.09 (0.01-0.34) and in MCD; IL-2: 0.25 (0.04-0.84), IL-12: 0.12 (0.02-0.42), GM-CSF: 0.41 (0.07-1.05), INF-γ: 0.14 (0.01-0.51), TNF-α: 0.09 (0.01-0.36).

Urinary levels (pg/mg Ucr) of Th2 cytokines in FSGS were; IL-4: 0.12 (0.02-0.43), IL-5: 0.11 (0.02-0.38), IL-10: 0.63 (0.16-1.8), IL-13: 0.11 (0.02-0.35) and in MCD; IL-4: 0.11 (0.01-0.42), IL-5: 0.12 (0.01-0.4), IL-10: 0.74 (0.04-2.6), IL-13: 0.11 (0.01-0.38).

No significant differences in urinary excretion of either Th1 or Th2 cytokines were evident between the two diseases (Figure 1).

**Figure 2:**
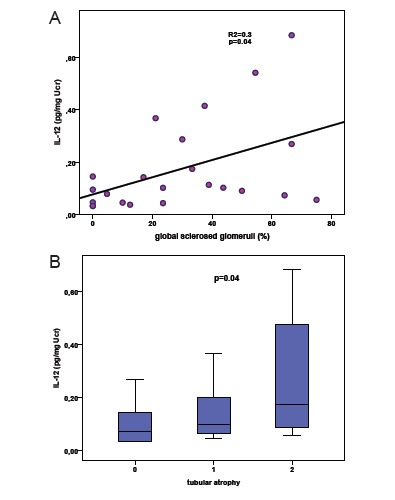
Correlation of histology with cytokine excretion in FSGS.

In FSGS, almost all cytokines had significant positive correlation with the degree of global sclerosis and severity of tubular atrophy (Table 2). In multivariate analysis IL-12 was the only independent factor correlating with both histological lesions, as shown in Figure 2.

**Table 2:** Correlations of cytokine excretion and histology in FSGS patients

	**Global sclerosis (%)**		**Tubular atrophy**	
	**r**	**P**	**r**	**P**
Th1 cytokines				
IL-2	0.5	0.02	0.4	0.05
IL-12	0.5	0.02*	0.4	0.04**
GM-CSF	0.5	0.01	0.2	0.2
INF-γ	0.5	0.01	0.4	0.04
TNF-α	0.5	0.02	0.4	0.07
Th2 cytokines				
IL-4	0.5	0.01	0.4	0.06
IL-5	0.5	0.01	0.5	0.02
IL-10	0.6	0.004	0.4	0.1
IL-13	0.4	0.03	0.4	0.08

### 4.3. Urinary cytokines and clinical parameters at presentation

**Figure 3:**
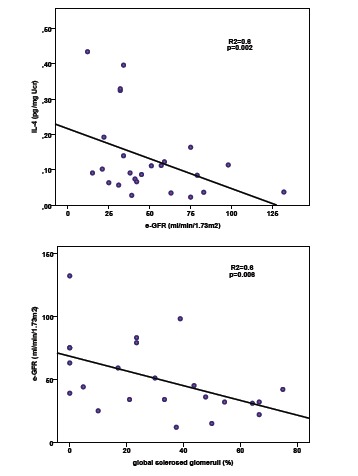
FSGS: Renal function at diagnosis, correlation with IL-4 urinary excretion and globally sclerosed glomeruli.

In FSGS eGFR (1) at presentation had significant negative correlation with the percentage of globally sclerosed glomeruli (r = -0.6, *P* < 0.001), the severity of tubular atrophy (r = -0.5, *P* = 0.01), degree of interstitial infiltration (r = -0.5, *P* = 0.02), urinary levels of IL-12 (r = -0.5, *P* = 0.02), INF-γ (r = -0.4, *P* = 0.04), IL-4 (r = -0.5, *P* = 0.02), IL-5 (r = -0.4, *P* = 0.04) and IL-13 (r = -0.4, *P* = 0.04). In multivariate analysis however, the only independent factors were the percentage of globally sclerosed glomeruli (r^2^= 0.4, *P* = 0.005) and urinary excretion of IL-4 (r^2^= 0.6, *P* = 0.002) (Figure 3). The severity of proteinuria, at presentation or at the end of follow up, did not have any significant correlation with the urinary excretion of cytokines.

In MCD, eGFR at presentation correlated only with the percentage of global sclerosis (r = -0.6, *P* = 0.02) and with the urinary excretion of GM-CSF (r = 0.6, *P* = 0.03).

### 4.4. Outcome of renal function

**Figure 4:**
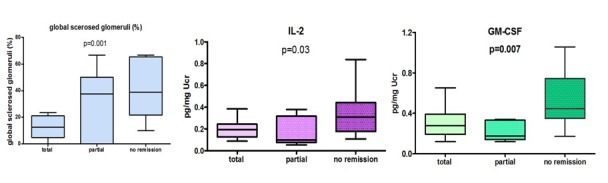
Outcome of renal function in FSGS patients, correlation with histology and urinary cytokines

Patients with FSGS were followed for 48.5 (16-108) months. At the end of this period 13/36 (36%) showed reduction in proteinuria and improvement in renal function and serum albumin levels, and according to KDIGO clinical practice guidelines for glomerulonephritis, were characterised as being in complete remission; 6/36 (17%) were in partial remission and 17/36 (47%) did not respond to treatment. Eleven patients (30.5%) reached end stage renal disease and commenced on hemodialysis. Patients with complete or partial remission had significantly lower urinary levels of IL-2, GM-CSF at presentation, and lower number of globally sclerosed glomeruli in renal biopsy, compared to those who did not respond (Figure 4).

During follow up mean eGFR was reduced significantly, eGFR (2) was 33.1 ± 29 mL/min/1.73 m^2^ vs. eGFR (1) 48.7 ± 27 mL/min/1.73 m^2^, *P* < 0.0001. eGFR (2) had significant correlation with renal function at diagnosis (eGFR 1), (r = 0.8, *P* < 0.001), percentage of globally sclerosed glomeruli (r = -0.6, p=0.001), degree of tubular atrophy (r=-0.5, *P* = 0.008), urinary levels of IL-2 (r = -0.4, *P* = 0.02), IL-12 (r = -0.4, *P* = 0.01), GM-CSF (r = -0.4, *P* = 0.02), INF-γ (r = -0.3, *P* = 0.04), IL-4 (r = -0.4, *P* = 0.05) and IL-10 (r = -0.4, *P* = 0.02). In multiple regression analysis, the percentage of globally sclerosed glomeruli was the only independent factor correlated with eGFR (2) (r^2^ = 0.5, *P* = 0.003).

**Figure 5:**
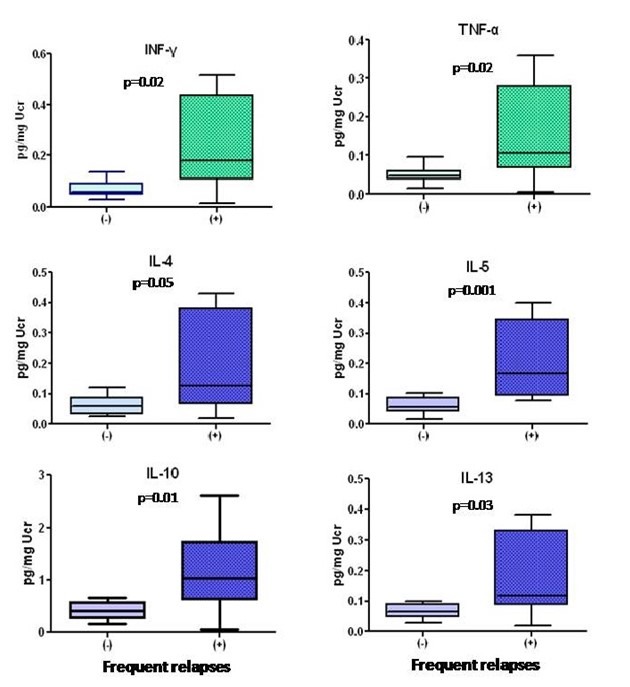
Relapses of nephrotic syndrome in MCN patients, correlation with urinary cytokines.

The outcome of renal function among the 21 patients with MCD was significantly better. Only one patient, who presented with acute renal failure requiring hemodialysis and had severe acute tubular necrosis in renal biopsy, did not respond to treatment and remained on dialysis. All other 20 patients responded, and after a follow up of 70 (12-120) months their eGFR (2) was 73.7 ± 28 vs. eGFR (1) 84.4 ± 30, *P* = NS. Eight patients (38%) had more than two relapses of nephrotic syndrome during follow up. These 8 patients with frequent relapses had significantly increased urinary levels of all Th2 cytokines (IL-4, IL-5, IL-10, IL-13) and also INF-γ and TNF-α (Figure 5).

## 5. Discussion

FSGS and MCD are glomerular diseases characterized by common clinical symptoms, such as heavy proteinuria or nephrotic syndrome, common histological findings, such as podocyte injury with no signs of proliferative disorders, but FSGS usually progresses to severe renal injury with glomerulosclerosis, tubulointerstitial fibrosis and arteriosclerosis, while histology in MCD alleviates after steroid treatment and full recovery is almost exclusively anticipated (3,6).

Why these two entities, sharing so many common clinical and histological characteristics prove to be so divergent in outcome? What should be the difference, in pathogenesis or during disease progress? These are questions usually set by clinicians but even today our knowledge is still incomplete to give sufficient answers. Cytokines produced by resident or infiltrated cells are proved to play major role in the pathogenesis and progression of primary and secondary glomerulopathies, by activating immunological responses and leading to renal tissue damage (9,13). Their urinary excretion has been demonstrated to represent their local production within the kidneys in many different types of glomerulonephritis. Based on these factors, we decided to evaluate urinary cytokine excretion of two groups of cytokines, produced mainly by two competitive T cell types, Th1 and Th2, and having different effects. INF-γ, IL-2, IL-12, TNF-α, GM-CSF are the main representatives of Th1 cytokines. Th1, as pre-inflammatory cytokines, activate macrophages, neutrophils, NKCs and memory cells. Th2 cytokines, including IL-4, IL-5, IL-10, IL-13, are anti-inflammatory molecules, they activate eosinophils and B-cells, and lead to antibody production (14-16).

In our study, there was no significant difference between FSGS and MCD in urinary excretion of the cytokines measured. In both types of glomerulopathy, there were increased cytokine urinary levels compared to controls, but not statistically significant. Many efforts have been made to find biomarkers distinguishing FSGS from MCD. suPAR is a molecule connected with the attractive theory of being implicated in the pathogenesis of FSGS as a permeability factor also causing recurrence of disease after transplantation. suPAR also seemed to discriminate FSGS from other primary diseases, even from MCD (18). However, repeated research gave conflicted results (19), and the Nephrotic Syndrome Study Network (NEPTUNE) showed that suPAR levels were correlated with renal function impairment in several primary glomerular diseases, and were not associated with FSGS after adjustment of eGFR (20). Other substances such as CD80 and MMP9 have recently been proposed as potentially differentiate FSGS from MCD (9,16). We have found significantly reduced urinary levels of EGF in patients with FSGS compared to MCD, but this needs further evaluation, as EGF is produced by tubular epithelial cells, and its reduction may only represent advanced tubular atrophy (10). Until now there is no definite marker in urine or serum which can differentiate the two entities.

However, the remarkable finding in this study was the substantial implication of Th1 cytokines in the pathogenesis and progression of FSGS, while in MCD Th2 cytokines seemed to have an essential role in the recurrent episodes of nephrotic syndrome. The severity of renal impairment at presentation in FSGS patients correlated with degree of global sclerosis and tubulointerstitial changes and urinary excretion of IL-12, INF-γ, IL-4, IL-5 and IL-13, with IL-4 and degree of global sclerosis being significant in multiple regression analysis. At the end of follow up, although multiple factors seemed to be correlated with the degree of renal function, only the severity of global sclerosis and urinary levels of IL-2 and GM-CSF were the independent significantly important parameters for the response to treatment and outcome of renal function. Multiple regression analysis showed that the only independent parameter for the main histological lesions, global sclerosis and tubular atrophy, was IL-12 urinary excretion. Interestingly, although IL-4, a Th2 cytokine, plays the main role in the early phase of the disease, Th1 cytokines, IL-12, IL-2 and GM-CSF constitute the main factors associated with histology and disease outcome. There are conflicting results about the pathogenetic effects of Th1/Th2 cytokines in FSGS. For many investigators FSGS was considered a Th2 mediated disease, but there is a growing dispute about this theory. Administration of IL-2/IL-2Ab in adriamycin induced nephropathy in mice can cause Treg expansion, leading to alleviation of proteinuria and renal damage, however, this result was not confirmed in humans. IL-2 administration was unable to reduce proteinuria in patients with FSGS (21,22). During the last years, there is accumulating evidence that IL-2 cytokine increases proteinuria, by means of in vitro studies showing that IL-2 induces albumin leakage across a podocyte monolayer, and in vivo studies indicating that activation through the IL-2R expressed on murine podocytes can cause podocyte injury (24). Furthermore, up-regulation of IL-2 and IL-2 receptor synthesis was evident in experimental models of two-thirds nephrectomised rats and tacrolimus treatment reduced degree of proteinuria by inhibiting IL-2 synthesis (23).

Furthermore, reduced levels of IL-2(+)CD3(+) and IFN-γ(+)CD3(+) in patients with FSGS were correlated with response to rituximab and were recently suggested as markers of beneficial outcome of the disease, supporting the results from our study (25).

There is not much information in the literature about the role of IL-2 in FSGS, but the knowledge about GM-CSF is even less. There is some evidence for the pathogenic role of M-CSF in the inflammatory (LPS-induced) nephritis, experimental model for ANCA associated vasculitis (AAV) and for the monocyte recruitment in AAV and lupus nephritis (LN) (26,27).

Recently, increased serum M-CSF levels have been associated with active vasculitis in humans and have been proposed as a biomarker of renal involvement of AAV, as well as serum and urine CSF-1 levels correlated with lupus activity and can potentially act as lupus biomarkers and predict the onset and recurrence of LN. M-CSF and CSF1 are produced by tubular epithelial cells, and implicated in macrophage recruitment and pathogenesis of inflammatory changes (28-30).

GM-CSF is derived from inflammatory and renal parenchymal cells and mediate proteinuria, crescent formation and tubular changes, leading to renal dysfunction in murine crescentic glomerulonephritis (31).

To our knowledge, this is the first evidence that GM-CSF urinary levels in FSGS are correlated with histology and could predict renal function outcome as an independent parameter. Pathogenetic mechanisms are not yet elucidated, but macrophage accumulation which follows GM-CSF excretion, represents a possible pathway (28-30).

Regarding the MCD patients, our findings suggest an interesting role of Th2 cytokines in the outcome of disease, and particularly a strong association of increased urinary levels of Th2 cytokines at time of renal biopsy, with recurrent episodes of relapse during follow up. Association of MCD with allergic reactions is well-known, as is the shift to Th2 type immune response, especially in atopic children with MCD (32,33). IL-4 and IL-13 are increased during active phase of the disease. Some investigators have also described increased serum levels of IL-4, IL-13 and IgE in MCD regardless active or remission phases (34). These cytokines, and in general Th2 shift immune response is connected to atopic reactions, or other diseases, such as Hodgkin disease, also associated with MCD, suggestive a pathogenetic relationship between Th2 cytokines and MCD. IL-13 has recently been implicated in the up-regulation of B7-1 and downregulation of nephrin and podocin expression of podocytes in WKY rats, leading to proteinuria and MCD (35).

## Conclusions

In conclusion, we did not find differences in the urinary levels of Th1 and Th2 cytokines between FSGS and MCD, but the cytokines have different roles in these two diseases. Elevated IL-2 and GM-CSF urinary levels could predict deterioration of renal function in FSGS, while increased Th2 cytokines in MCD were associated with frequent relapses in MCD.

## Limitations of the study

This is a single center study with a limited proportion of patients.

## Conflicts of Interest

Authors declare no conflict of interests.

## Authors’ contribution

MaS, MiS, DVD and TK conducted the study. ES and ITL analyzed the data. AP analyzed the renal biopsies and put the diagnosis. MaS and AP performed the laboratory methods. MiS wrote the paper. AP and GE made corrections and edited the final draft. All authors signed the manuscript.

## Funding/Support

The authors contributed to the funding of this research.
